# Supplementary material for: Multi‐modal meta‐analysis of cancer cell line omics profiles identifies ECHDC1 as a novel breast tumor suppressor
Source: Mol Syst Biol. 2021 Mar 22;17(3):e9526. doi: 10.15252/msb.20209526 (PMC7983037; doi:10.15252/msb.20209526)
Supplement: Supplementary file 1 — Appendix [file MSB-17-e9526-s011.pdf]

Tables of contents

Appendix Figure S1
Appendix Figure S2
Appendix Figure S3
Appendix Figure S4
Appendix Figure S5
Appendix Figure S6
Appendix Figure S7
Appendix Figure S8
Appendix Figure S9
Appendix Figure S10
Appendix Figure S11
Appendix Figure S12
Appendix Figure S13
Appendix Figure S14
Appendix Figure S15
Appendix Figure S16
Appendix Figure S17
Appendix Figure S18
Appendix Figure S19

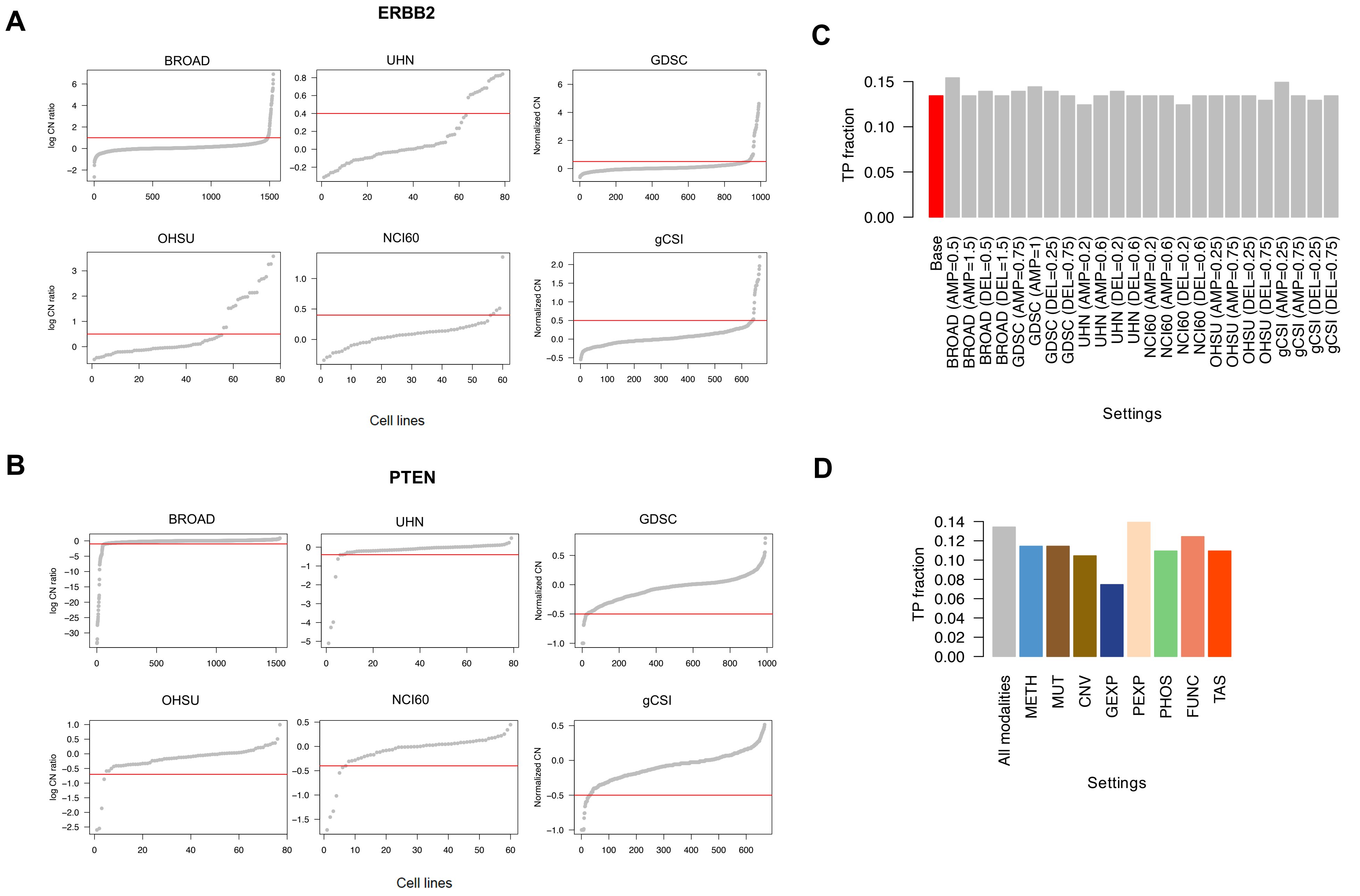

Appendix Figure S1: Copy number distribution of two well-known CNV alterations in breast tumors; (A) ERBB2 is a known to be frequent amplification, and (B) PTEN is known to be a frequent deletion. We selected for the CLIP meta-analysis the threshold for calling amplifications and deletions in each dataset at the value where we observed a sharp deflection (red horizontal line). (C) Benchmarking the performance of CLIP to identify well-known breast cancer driver genes. True positive (TP) fraction of unique cancer driver genes (n=201) for the three defined breast cancer subtypes as identified by CLIP at various threshold for defining copy number deletions (DEL) and amplifications (AMP) in each individual dataset. At CLIP base setting, the thresholds are: (i) for calling AMP – BROAD >=1; GDSC, gCSI and OHSU >= 0.5; and UHN, NCI60 >= 0.4, (ii) for calling DEL - BROAD <1; GDSC, gCSI and OHSU < 0.5; and UHN, NCI60 < 0.4. All other settings are variations over the CLIP setting with altered threshold for the specified dataset. (D) Assessment of the contribution of different data modalities to the performance of CLIP to identify well-known breast cancer driver genes. True positive (TP) fraction of unique cancer driver genes (n=201) for the three defined breast cancer subtypes as identified by CLIP at the base setting in which all the modalities were included. The other bars show the performance under a setting in which each data modality was removed from the CLIP pipeline one at a time. Lower consistency within datasets of PEXP modality may explain the improved performance under the removal of PEXP modality

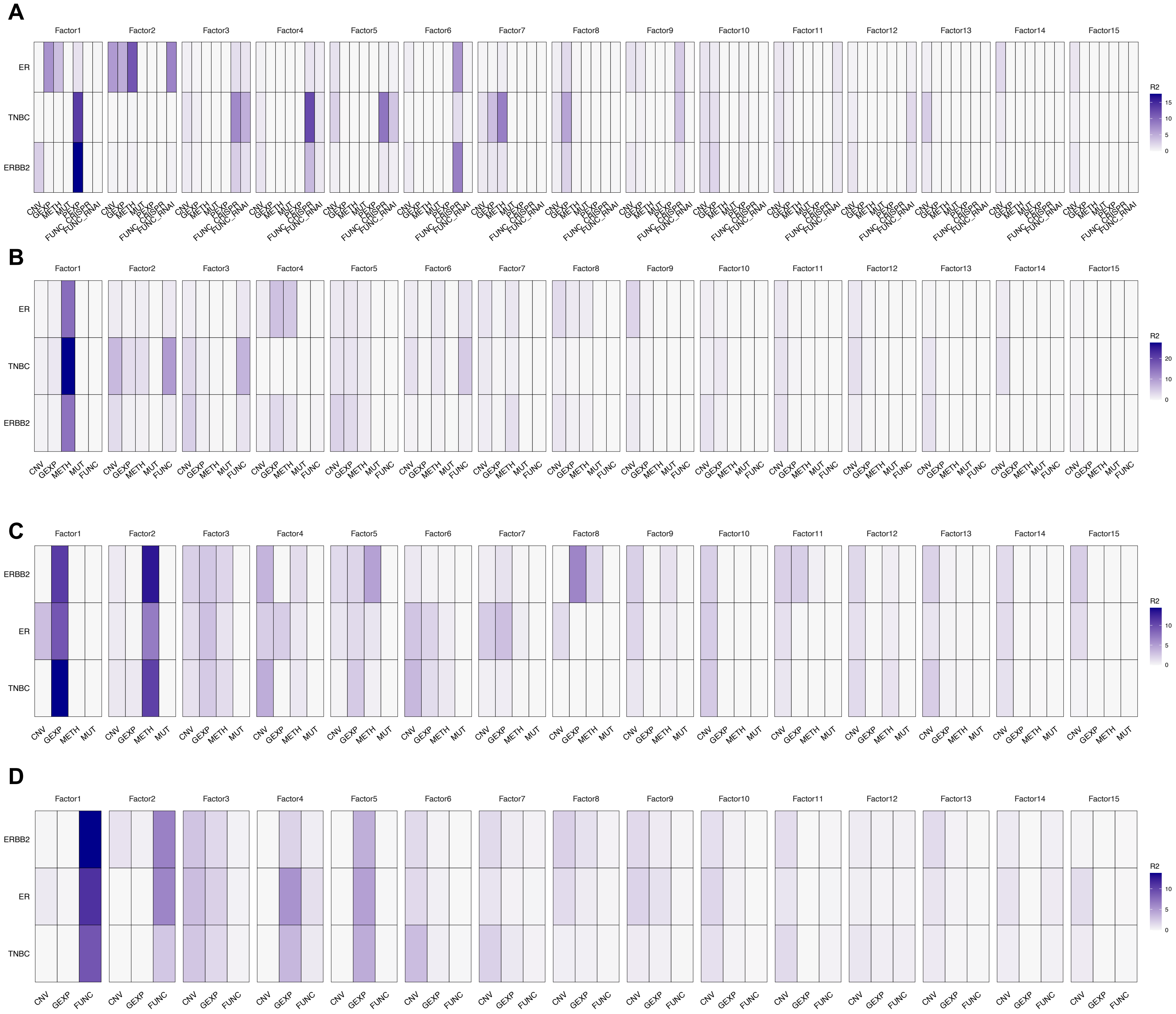

Appendix Figure S2: Proportion of variance explained for each breast cancer subtype by each data modality for 15 latent factors in MOFA+. Views and factors with the highest variance were selected further, and the top-20 genes loading on to each factor were checked for overlap with known cancer driver genes; (A) BROAD_CCLE (B) GDSC (C) OHSU and (D) UHN. METH, gene promoter methylation; MUT, gene point mutation; CNV, copy number variation; GEXP, gene mRNA expression; PEXP, protein expression; PHOS, protein phosphorylation; FUNC, gene dependency; DSS, drug sensitivity; TAS, protein addiction.

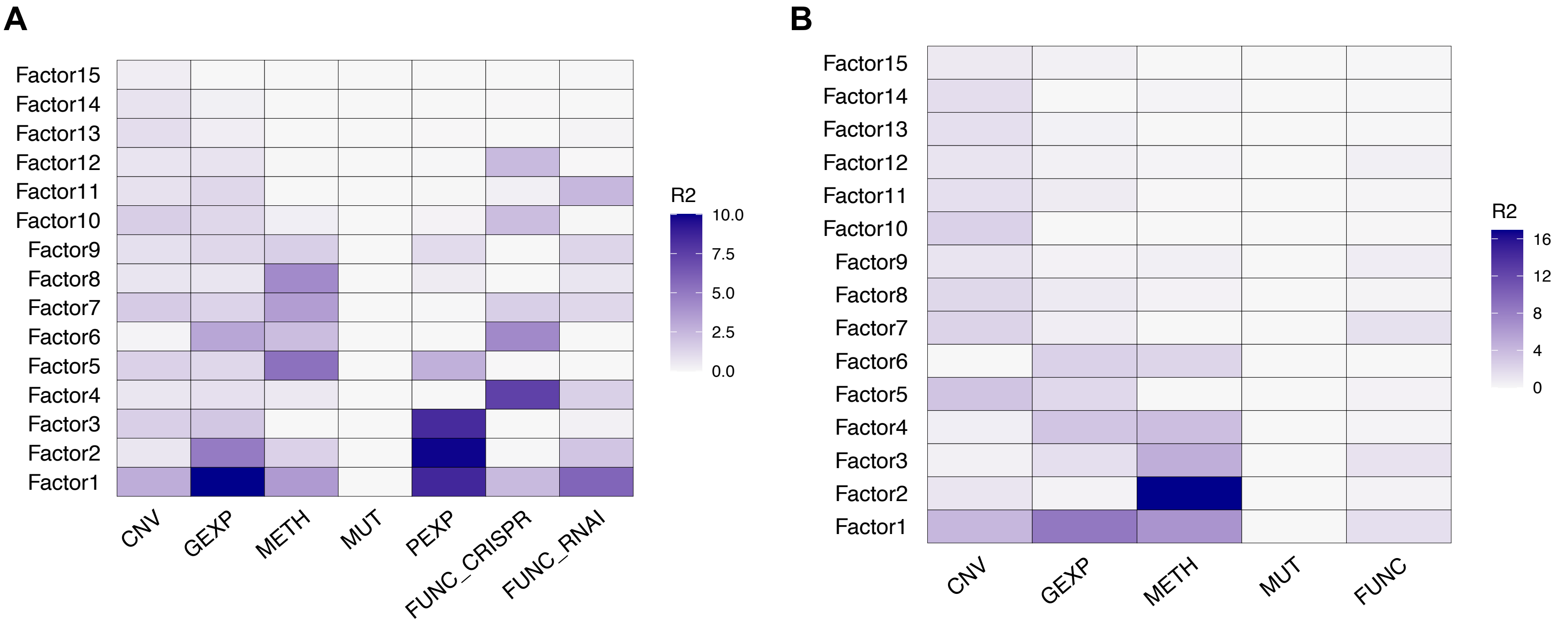

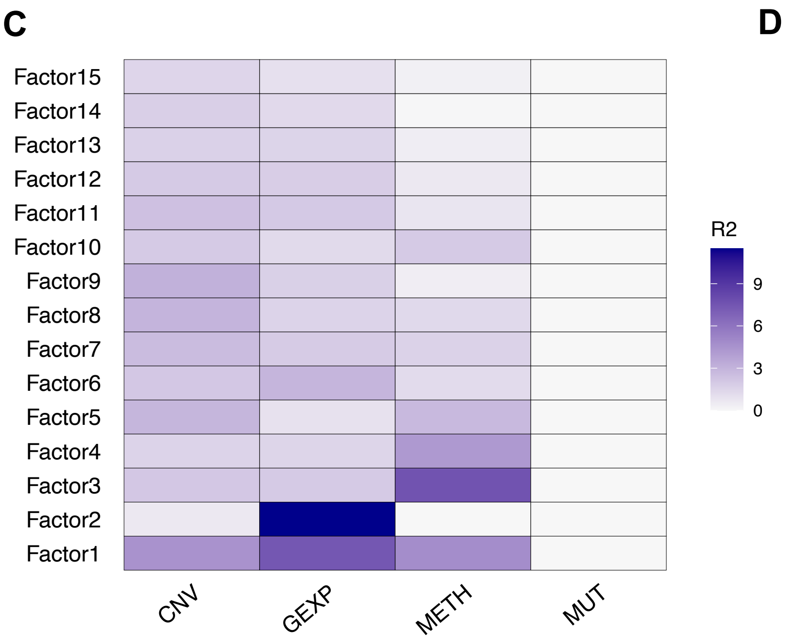

D

	CCLE		SANGER		OHSU	
	METH	GEXP	METH	GEXP	METH	GEXP
**Factor1**	12842	17548	1053	435		16169
**Factor2**	421	5849	9877	7923		12688
**Factor3**	12135	16951	998	670		9132
**Factor4**	1646	5589	12633	11206		1925
**Factor5**	12330	16709	4001	306		14059
**Factor6**	11612	14607	11321	11557		9506
**Factor7**	1164	3257	7975	7425		5744
**Factor8**	4498	13316	9969	659		14667
**Factor9**	2586	3815	7104	13197		2782
**Factor10**	10486	16436	5679	402		10869
**Factor11**	12561	15442	5993	4206		12094
**Factor12**	3907	5669	7803	7837		15437
**Factor13**	8267	7242	4385	8534		2305
**Factor14**	3104	7867	11314	9222		8335
**Factor15**	5450	4898	4137	4765		10081

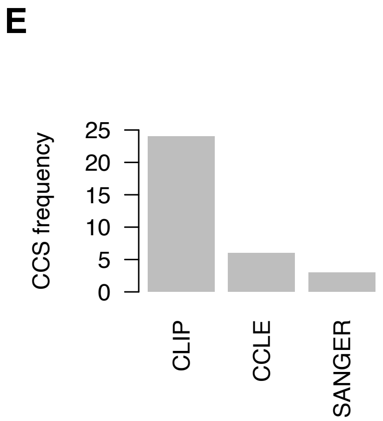

Appendix Figure S3: Proportion of variance explained for each breast cancer subtype by each data modality for 15 latent factors in MOFA+. For GEXP and METH views, factors with highest variance were then selected further and checked if ECHDC1 was one of the top-20 genes loading on to each factor; (A) BROAD_CCLE (B) GDSC (C) OHSU. (D) Ranks of the weight scores of ECHDC1 loading on to each factor in every dataset for GEXP and METH modalities. Ranking of weight score was done from positive to negative for METH, and the reverse direction for GEXP. ECHDC1 methylation levels were not measured in OSHU dataset. (E) Frequency of ECHDC1 being identified as an outlier gene, i.e. a Cancer Cell line Specific (CCS_ gene by CLIP and in each individual dataset using a simpler outlier detection based approach (see Methods). METH, gene promoter methylation; MUT, gene point mutation; CNV, copy number variation; GEXP, gene mRNA expression; PEXP, protein expression; PHOS, protein phosphorylation; FUNC, gene dependency.

A

METHYLATION (METH)				
	BROAD_CCLE	SANGER	OHSU	NCI60
**Number of cell lines**	843	1026	55	60
**Details of processed data provided by authors of study**	• Averaged gene-level methylation profiles of promoters situated 1 kb upstream of transcription start<br>• Data generated using reduced representation bisulfite sequencing (RRBS) method	• Probe intensities generated by Illumina HumanMethylation450 BeadChip	• Probe intensities generated by Illumina Infinium Human Methylation27 BeadChip	• Probe intensities generated by Illumina Infinium Human Methylation27 BeadChip
**Pre-processing steps (conducted in this study)**		1. Quality control was performed by removing CpG sites with missing rate >5% and detection P > 0.05.<br>2. Gene level methylation intensities were obtained by averaging the methylation levels of CpG sites located in the annotated promoter site of each gene within a range of 200 to 1500 base pairs (bp) upstream of the transcription start site	1. Quality control was performed by removing CpG sites with missing rate >5% and detection P > 0.05.<br>2. Gene level methylation intensities were obtained by averaging the methylation levels of CpG sites located in the annotated promoter site of each gene within a range of 200 to 1500 base pairs (bp) upstream of the transcription start site	1. Quality control was performed by removing CpG sites with missing rate >5% and detection P > 0.05.<br>2. Gene level methylation intensities were obtained by averaging the methylation levels of CpG sites located in the annotated promoter site of each gene within a range of 200 to 1500 base pairs (bp) upstream of the transcription start site
**Correlation analyses**	• Spearman's Correlation Coefficient			
**CLIP Input pre-processing (conducted in this study)**	1. Subset to human coding genes<br>2. Quantile normalization on gene level methylation profiles<br>3. Outlier Evidence Score (OES) scaling			

B

MUTATION (MUT)						
	BROAD_CCLE	SANGER	gCSI	UHN	OHSU	NCI60
**Number of cell lines**	1530	1000	675	81	50	60
**Details of processed data provided by authors of study**	• Point mutation annotations provided	• Point mutation annotations provided	• Point mutation annotations provided	• Unannotated point mutation list	• Unannotated point mutation list	• Point mutation annotations provided
**Pre-processing Steps (conducted in this study)**	1. Input data: Point mutation list per cell line<br>2. For annotated mutations, consider the following classifications as deleterious:<br>- isDeleterious = TRUE<br>- TCGA hotspot = TRUE<br>- COSMIC hotspot = TRUE<br>3. For unannotated mutations, annotate with FATHMM<br>4. Consider point mutations with FATHMM score $\leq$ -0.75 as deleterious<br>5. Gene level summarization by presence or absence of a point mutation	1. Input data: Point mutation list per cell line<br>2. For annotated mutations, consider the following classifications as deleterious:<br>- FATHMM prediction = pathogenic<br>3. For unannotated mutations, annotate with FATHMM<br>4. Consider point mutations with FATHMM score $\leq$ -0.75 as deleterious<br>5. Gene level summarization by presence or absence of a point mutation	1. Input data: Point mutation list per cell line<br>2. For annotated mutations, consider the following classifications as deleterious:<br>- SIFT = deleterious<br>- PolyPhen = probably-damaging<br>- Condel= deleterious<br>3. For unannotated mutations, annotate with FATHMM<br>4. Consider point mutations with FATHMM score $\leq$ -0.75 as deleterious<br>5. Gene level summarization by presence or absence of a point mutation	1. Input data: Point mutation list per cell line<br>2. For unannotated mutations, annotate with FATHMM<br>3. Consider point mutations with FATHMM score $\leq$ -0.75 as deleterious<br>4. Gene level summarization by presence or absence of a point mutation	1. Input data: Point mutation list per cell line<br>2. For unannotated mutations, annotate with FATHMM<br>3. Consider point mutations with FATHMM score $\leq$ -0.75 as deleterious<br>4. Gene level summarization by presence or absence of a point mutation	1. Input data: Point mutation list per cell line<br>2. For annotated mutations, consider the following classifications as deleterious:<br>- SIFT = deleterious<br>- PolyPhen = probably-damaging<br>- MA FI Impact = high or medium<br>- Cancer relevant<br>3. For unannotated mutations, annotate with FATHMM<br>4. Consider point mutations with FATHMM score $\leq$ -0.75 as deleterious<br>5. Gene level summarization by presence or absence of a point mutation
**Correlation analyses**	• Matthew's Correlation Coefficient					
**CLIP Input pre-processing (conducted in this study)**	1. Proportion Scores (PS) were generated. PS is the proportion of cell lines in which the particular genomic alteration is observed					

C

COPY NUMBER VARIATION (CNV)						
	BROAD_CCLE	SANGER	gCSI	UHN	OHSU	NCI60
**Number of cell lines**	1532	991	667	79	77	60
**Details of processed data provided by authors of study**	• Data generated by Affymetrix SNP 6.0 arrays<br>• Copy numbers were then inferred using the circular binary segmentation (CBS) algorithm.<br>• Gene level copy number ratios were provided.	• Data generated by Affymetrix SNP 6.0 arrays were processed with the PICNIC algorithm<br>• Total copy number calls made were normalized by the ploidy levels.<br>• Gene level copy number intensities were provided.	• Data generated by Illumina HumanOmni2.5_4v1 arrays were processed with the PICNIC algorithm.<br>• Total copy number calls made were normalized by the ploidy levels.<br>• Gene level copy number intensities were provided.	• Data generated by Illumina Human Omni-Quad Bead chips arrays<br>• SNP array data were segmented by Circular Binary Segmentation (CBS).<br>• CBS segments intensities mapped to genes were provided	• Data generated by Affymetrix SNP 6.0 arrays<br>• Copy numbers were then inferred using the circular binary segmentation (CBS) algorithm.<br>• Gene level copy number ratios were provided.	• Gene level summarized log-scale intensities representing copy number profiles generated by combining probe intensities from four platforms (Agilent Human Genome CGH Microarray 44A, Nimblegen HG19 CGH 385K WG Tiling v2.0, Affymetrix GeneChip Human Mapping 500k Array Set and Illumina Human1Mv1_C BeadChip) were provided.
**Correlation analyses**	• Spearman's Correlation Coefficient					
**CLIP Input pre-processing (conducted in this study)**	1. Gene-level copy amplification and deletions were defined by +/- 1 cutoffs respectively.<br>2. Two binary matrices representing CNV_AMP and CNV_DEL calls were used as input for CLIP	1. Gene-level copy amplification and deletions were defined by +/- 0.5 cutoffs respectively.<br>2. Two binary matrices representing CNV_AMP and CNV_DEL calls were used as input for CLIP	1. Gene-level copy amplification and deletions were defined by +/- 0.5 cutoffs respectively.<br>2. Two binary matrices representing CNV_AMP and CNV_DEL calls were used as input for CLIP	1. Gene-level copy amplification and deletions were defined by +/- 0.4 cutoffs respectively.<br>2. Two binary matrices representing CNV_AMP and CNV_DEL calls were used as input for CLIP	1. Gene-level copy amplification and deletions were defined by +/- 0.5 cutoffs respectively.<br>2. Two binary matrices representing CNV_AMP and CNV_DEL calls were used as input for CLIP	1. Gene-level copy amplification and deletions were defined by +/- 0.4 cutoffs respectively.<br>2. Two binary matrices representing CNV_AMP and CNV_DEL calls were used as input for CLIP
	3. Subset to human coding genes<br>4. Proportion Scores (PS) were generated for each CNV binary call matrix. PS is the proportion of cell lines in which the particular genomic alteration is observed					

D

GENE EXPRESSION (GEXP)						
	BROAD_CCLE	GDSC	gCSI	UHN	OHSU	NCI60
**Number of cell lines**	1019	991	674	82	56	60
**Details of processed data provided by authors of study**	• Data generated by RNA sequencing<br>• Gene normalized RPKM units were used	• Data generated by Affymetrix Human Genome U219 array<br>• RMA normalized gene level intensities were used	• RPKM unit gene expression profile generated by RNA sequencing was used	• Gene normalized log2 FPKM units were used	• Gene normalized normalized RPKM units were used	• Gene level processed GCRMA normalized gene expression profiles generated with Affymetrix Human Genome U133 plus 2.0 array was used
**Pre-processing Steps (conducted in this study)**						Multiple probes for identical genes were averaged
**Correlation analyses**	• Spearman's Correlation Coefficient					
**CLIP Input pre-processing (conducted in this study)**	1. Subset to human coding genes<br>2. Quantile normalization<br>3. Outlier Evidence Score (OES) scaling					

E

PROTEIN EXPRESSION (PEXP)						
	BROAD_CCLE	SANGER	MGHCC_BREAST	MPIB_HGSOC	UW_TNBC	NCI60
**Number of cell lines**	375	50	41	30	20	59
**Details of processed data provided by authors of study**	• Proteomic profiles were generated by MS using TMT-labelled multiplexed protocol for sample preparation.<br>• Bridge normalized and non-bridge normalized data was obtained	• Multiplexed quantitative MS-based proteomics was performed by the isobaric labeling technology with tandem mass tag (TMT) reagents.<br>• Normalization step involved row-mean scaling of column-normalized intenstiies	• Multiplexing was performed using the isobaric labeling technology with ten-plex tandem mass tag (TMT) reagent.<br>• Bridge normalized intensties were obtained.	• Label-free quantitative mass-spectrometry-based proteomics technology was performed.<br>• LFQ estimates were used.	• Data generated by non-multiplexed label-free quantitative mass-spectrometry (MS)-based proteomics technology.<br>• label-free iBAQ based quantitative proteomic profiles were used.	• Label-free iBAQ based quantitative estimates of protein levels
**Pre-processing Steps (conducted in this study)**	Multiple peptides mapping to identical proteins were averaged	Multiple peptides mapping to identical proteins were averaged	Multiple peptides mapping to identical proteins were averaged	Multiple peptides mapping to identical proteins were averaged	Multiple peptides mapping to identical proteins were averaged	Multiple peptides mapping to identical proteins were averaged
**Correlation analyses**	• Spearman's Correlation Coefficient					
**CLIP Input pre-processing (conducted in this study)**	1. Quantile normalization<br>2. Outlier Evidence Score (OES) scaling					

F

PROTEIN PHOSPHORYLATION (PHOS)						
	BROAD_CCLE	SANGER	MCLP	UHN	OHSU	NCI60
**Number of cell lines**	899	50	650	77	52	60
**Details of processed data provided by authors of study**	• Data generated using reverse phase protein arrays (RPPA)<br>• Protein phosphorylation levels of 217 phosphosites were available	• Data generated using multiplexed quantitative mass-spectrometry (MS)-based proteomics	• Data generated using reverse phase protein arrays (RPPA)<br>• Protein phosphorylation levels of 382 proteins using 452 antibodies were available	• Data generated using reverse phase protein arrays (RPPA)<br>• Protein phosphorylation levels of 193 proteins using 245 antibodies were available	• Data generated using reverse phase protein arrays (RPPA)<br>• Protein phosphorylation levels of 146 proteins were available	• Data generated using reverse phase protein arrays (RPPA)<br>• Protein phosphorylation levels of 94 proteins were available
**Pre-processing Steps (conducted in this study)**	Multiple phosphosites mapping to identical proteins were averaged	Multiple phosphosites mapping to identical proteins were averaged	Multiple phosphosites mapping to identical proteins were averaged	Multiple phosphosites mapping to identical proteins were averaged	Multiple phosphosites mapping to identical proteins were averaged	Multiple phosphosites mapping to identical proteins were averaged
**Correlation analyses**	• Spearman's Correlation Coefficient					
**CLIP Input pre-processing (conducted in this study)**	1. Quantile normalization<br>2. Outlier Evidence Score (OES) scaling					

G

FUNCTIONAL (FUNC)							
	BROAD_ACHILLES	BROAD_AVANA	BROAD_GECKO	BROAD_AML	DRIVE	SANGER	UHN
**Number of cell lines**	501	485	33	14	397	325	120
**Details of processed data provided by authors of study**	• Data generated by genome-wide RNAi screens<br>• Processed using the DEMETER alrogithm	• Data generated by genome-wide CRISPR-Cas9 knockout screens using the Avana library<br>• Processed using the CERES alrogithm	• Data generated by genome-wide CRISPR-Cas9 knockout screens using the sgRNA GeCKO v2 library<br>• Processed using the CERES alrogithm	• Data generated by genome-wide CRISPR-Cas9 knockout screens using the Sabatini library<br>• Processed using the CERES alrogithm	• Data generated by genome-wide RNAi screens<br>• Processed using the DEMETER alrogithm	• Data generated by genome-wide CRISPR-Cas9 knockout screens using the Avana library<br>• Copy number bias-corrected gene dependency scores were used	• Data generated by genome-wide RNAi screens<br>• Processed using the DEMETER alrogithm
**Pre-processing Steps (conducted in this study)**	Multiple shRNAs mapping to identical gene were averaged				Multiple shRNAs mapping to identical gene were averaged		Multiple shRNAs mapping to identical gene were averaged
**Correlation analyses**	• Spearman's Correlation Coefficient						
**CLIP Input pre-processing (conducted in this study)**	1. Subset to human coding genes<br>2. Quantile normalization<br>3. Outlier Evidence Score (OES) scaling						

H

DRUG SENSITIVITY (DSS)						
	BROAD_CCLE	BROAD_CTRP	SANGER	gCSI	OHSU	FIMM
**Number of cell lines**	887	504	1072	409	71	50
**Details of processed data provided by authors of study**	• Drug sensitivity screens were based on CellTiter-Glo (CTG) assay to measure cell viabilities<br>• Data processed using DSS2 algorithm downloaded from PharmacoDB	• Drug sensitivity screens were based on CellTiter-Glo (CTG) assay to measure cell viabilities<br>• Data processed using DSS2 algorithm downloaded from PharmacoDB	• Drug sensitivity screens were based on fluorescent nucleic acid stain probe Syto 60 assay to measure cell viabilities<br>• Data processed using DSS2 algorithm downloaded from PharmacoDB	• Drug sensitivity screens were based on CellTiter-Glo (CTG) assay to measure cell viabilities<br>• Data processed using DSS2 algorithm downloaded from PharmacoDB	• Drug sensitivity screens were based on CellTiter-Glo (CTG) assay to measure cell viabilities<br>• Data processed using DSS2 algorithm downloaded from PharmacoDB	• Drug sensitivity screens were based on CellTiter-Glo (CTG) assay to measure cell viabilities<br>• Data processed using DSS2 algorithm downloaded from PharmacoDB
**Pre-processing Steps (conducted in this study)**						
**Correlation analyses**	• Spearman's Correlation Coefficient					
**CLIP Input pre-processing (conducted in this study)**	Not applicable					

I

TARGET ADDICTION SCORES (TAS)						
	BROAD_CCLE	BROAD_CTRP	SANGER	gCSI	OHSU	FIMM
**Number of cell lines**	887	504	1072	409	71	50
**Details of processed data provided by authors of study**	• Drug sensitivity screens were based on CellTiter-Glo (CTG) assay to measure cell viabilities<br>• Data processed using DSS2 algorithm downloaded from PharmacoDB	• Drug sensitivity screens were based on CellTiter-Glo (CTG) assay to measure cell viabilities<br>• Data processed using DSS2 algorithm downloaded from PharmacoDB	• Drug sensitivity screens were based on fluorescent nucleic acid stain probe Syto 60 assay to measure cell viabilities<br>• Data processed using DSS2 algorithm downloaded from PharmacoDB	• Drug sensitivity screens were based on CellTiter-Glo (CTG) assay to measure cell viabilities<br>• Data processed using DSS2 algorithm downloaded from PharmacoDB	• Drug sensitivity screens were based on CellTiter-Glo (CTG) assay to measure cell viabilities<br>• Data processed using DSS2 algorithm downloaded from PharmacoDB	• Drug sensitivity screens were based on CellTiter-Glo (CTG) assay to measure cell viabilities<br>• Data processed using DSS2 algorithm downloaded from PharmacoDB
**Pre-processing Steps (conducted in this study)**	1. Drug sensitivity data processed using Target Addiction Score (TAS) algorithm<br>2. Drug target data extracted from Drug Target Commons database					
**Correlation analyses**	• Spearman's Correlation Coefficient					
**CLIP Input pre-processing (conducted in this study)**	1. Subset target set to human coding genes<br>2. Quantile normalization<br>3. Outlier Evidence Score (OES) scaling					

Appendix Figure S4: Details of the pre-processing steps applied to every dataset considered in the current study. See Methods section for more details of the preprocessing steps.

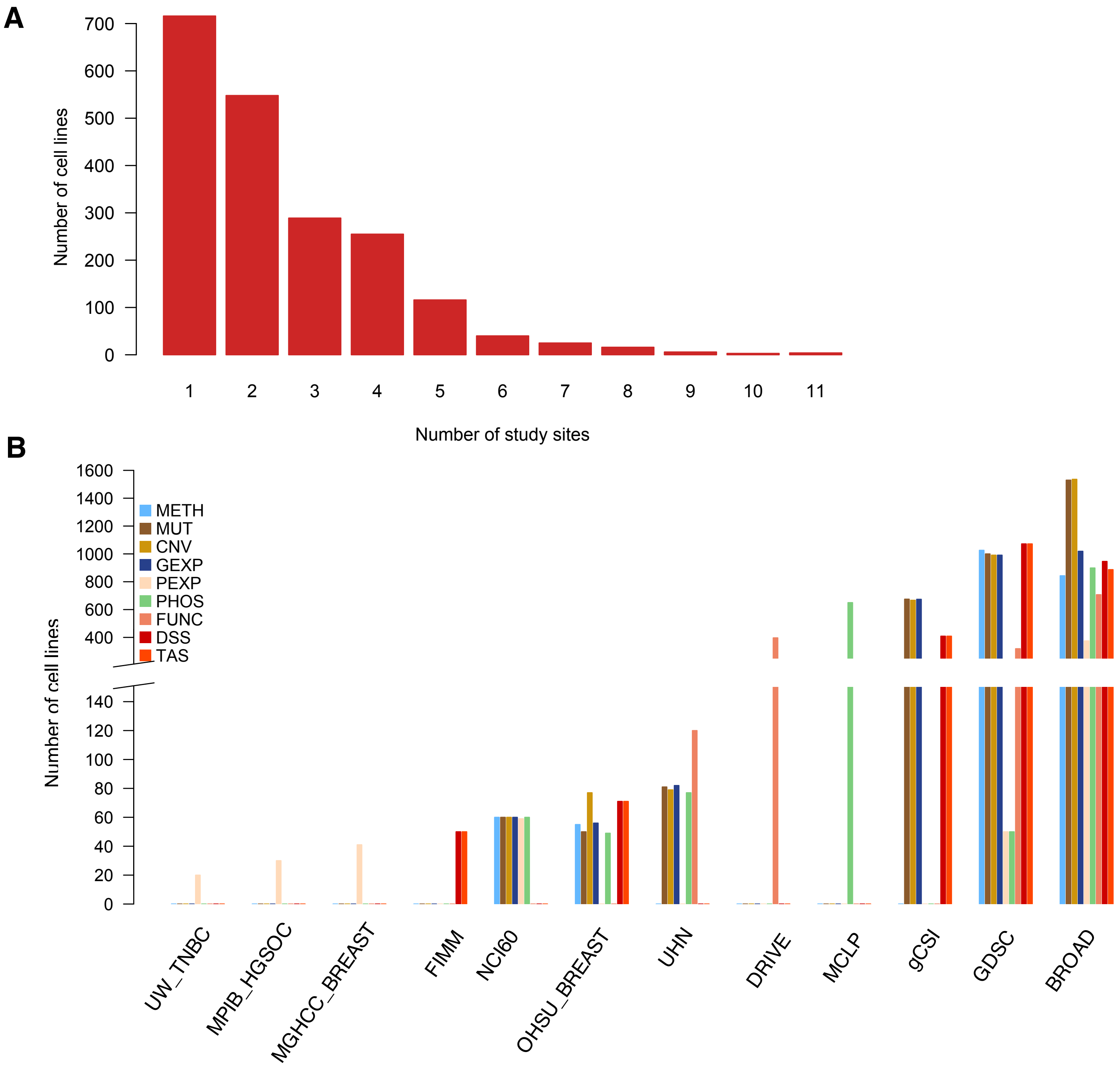

Appendix Figure S5: (A) The number of cell lines having available data from the 12 different research sites that were considered in the study. (B) The number of cell lines having available data at each research site by data modality type. METH, gene promoter methylation; MUT, gene point mutation; CNV, copy number variation; GEXP, gene mRNA expression; PEXP, protein expression; PHOS, protein phosphorylation; FUNC, gene dependency; DSS, Drug sensitivity; TAS, protein addiction.

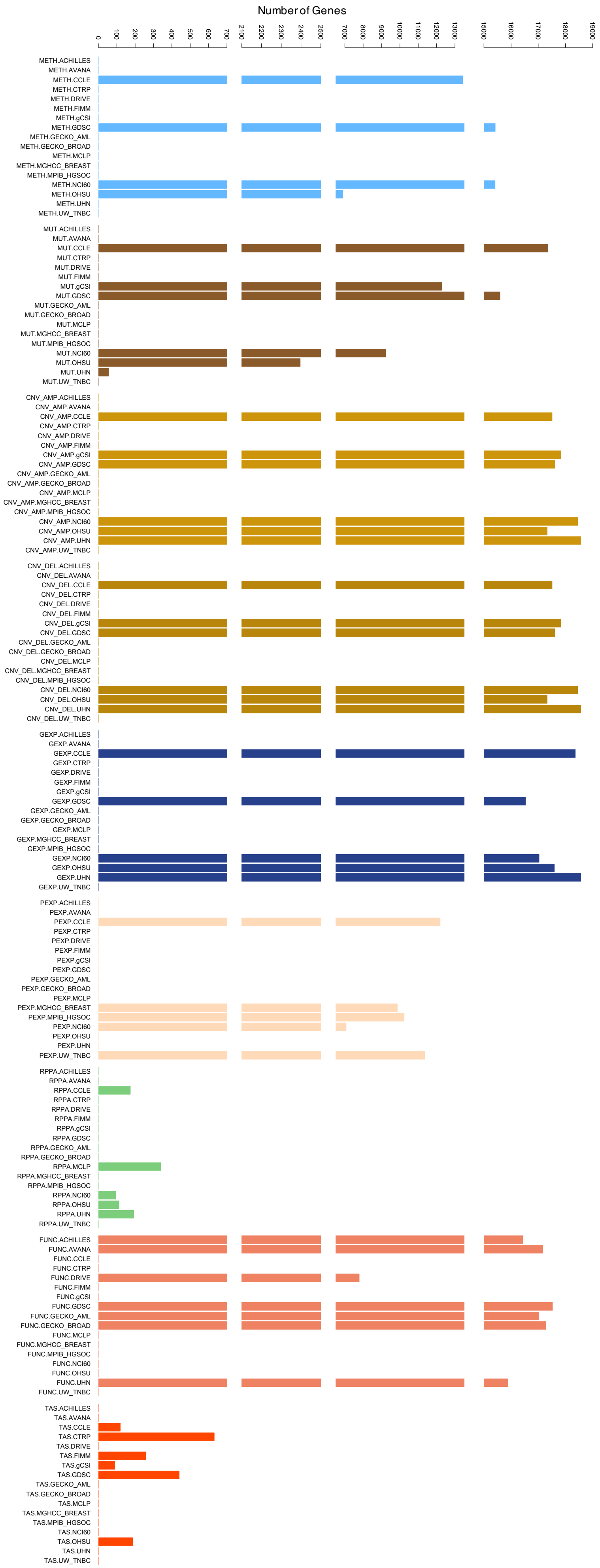

Appendix Figure S6: Breakdown of number of genes for which data was available for each modality at each research site. For CNV modality, the datasets were categorized into amplifications and deletions, namely CNV_AMP and CNV_DELs, which were included in the CLIP framework. METH, gene promoter methylation; MUT, gene point mutation; CNV, copy number variation; GEXP, gene mRNA expression; PEXP, protein expression; PHOS, protein phosphorylation; FUNC, gene dependency; DSS, Drug sensitivity; TAS, protein addiction.

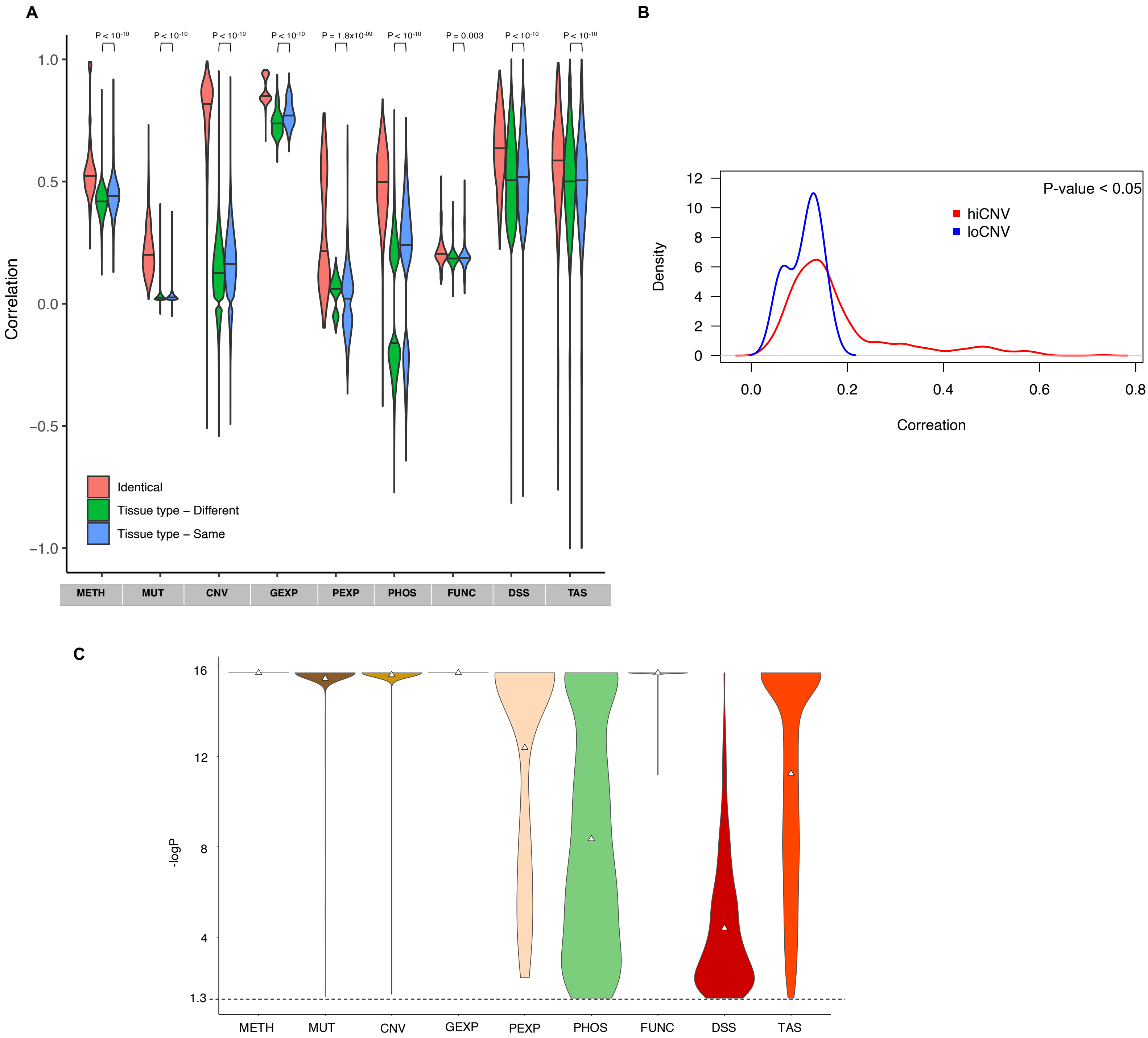

Appendix Figure S7: (A) Correlation of the data modalities of cancer cell lines profiled at multiple research sites. Spearman correlation was calculated for the shared set of genes between identical cell lines and non-identical cell lines, and further categorized by tissue identity (i.e. whether the cell lines comes from the same tissue-of-origin). Statistical comparison was done with the Wilcoxon test. (B) Distribution of correlation of mutational profiles of identical cell lines categorized into low CNV correlation (loCNV) group [$r^2_{CNV} < 0.1$] and high CNV (hiCNV) group [$r^2_{CNV} >= 0.1$]. P-value was calculated with the Wilcoxon test. (C) Negative logarithm of the P-values (-logP) calculated for all the correlations among identical cell lines between research sites to study the effect of the sample size (number of genes N_g) on the estimated correlations. P-values < 2.0×10^{-16} were set to 2×10^{-16}. The dotted line indicates the significance cut-off of P=0.05. The triangle symbol indicates mean P-value.

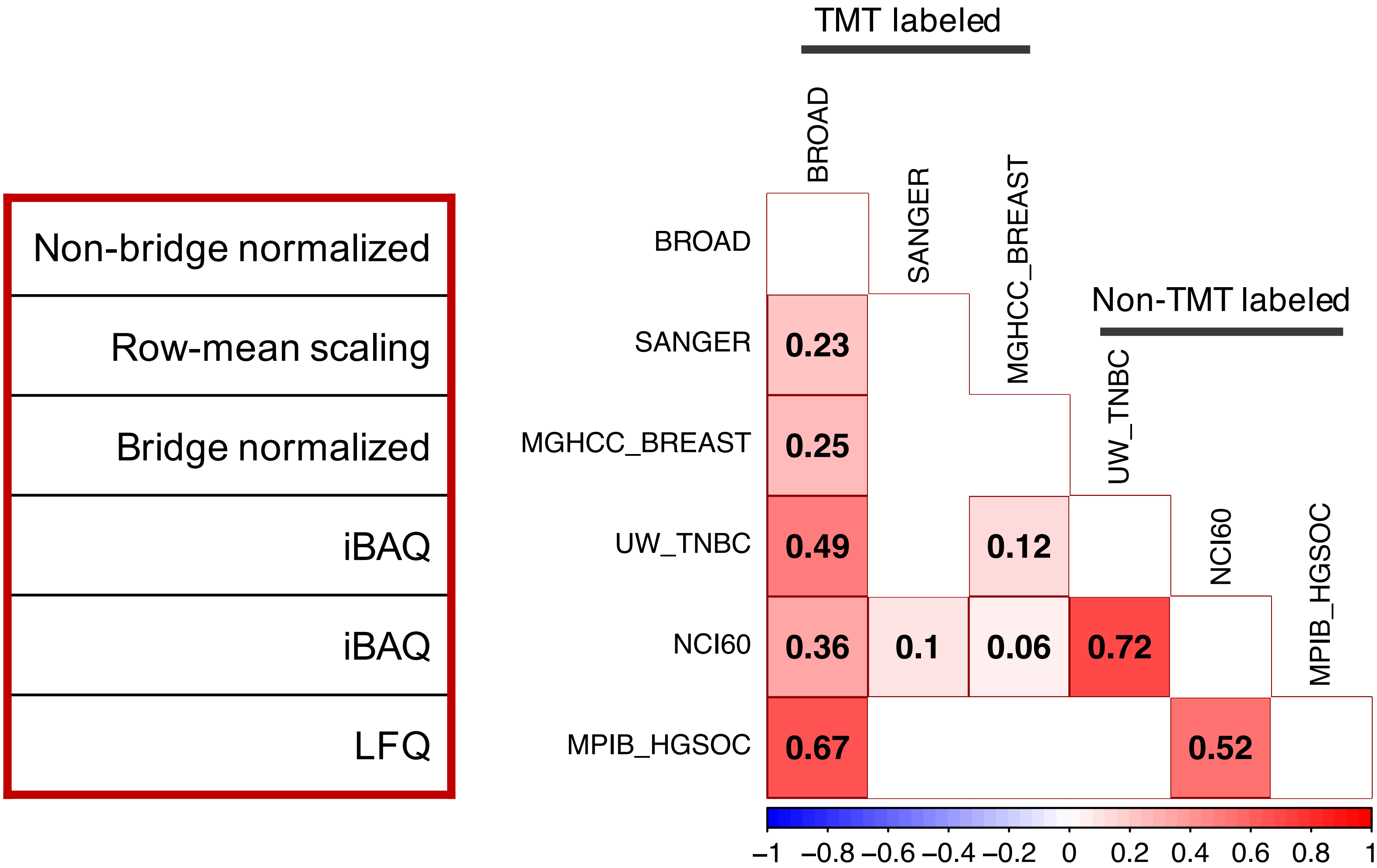

Appendix Figure S9: Contribution of data normalization methods (left) to the reproducibility of MS-based proteomic profiles (right). The correlation matrix shows average Spearman correlation of MS-based proteomic profiles between different study sites generated using different peptide labeling procedures and normalization methods. The empty cells indicate that no identical cell lines were profiled between the two datasets. Number of overlapping cell lines between any two datasets used for estimating the average correlation metrics ranges between 3 – 27 with a mean of 7.8.

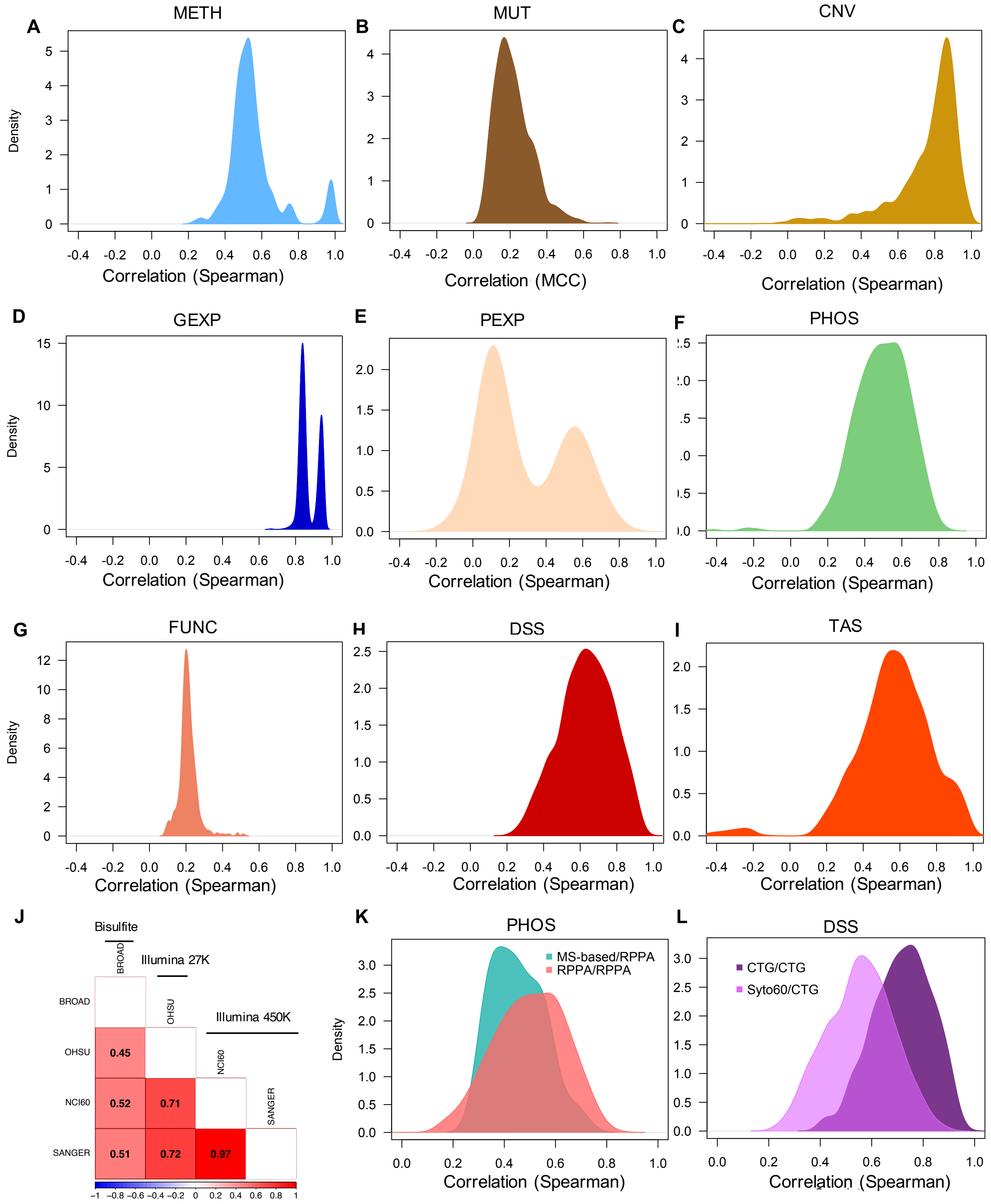

Appendix Figure S8: (A-I) Density plot of Spearman correlation coefficients of identical cell lines between multiple study sites for each data modality. The presence of bi-modality indicates a major technical or biological factor contributing to the mixed distributions. (J) Average spearman correlation of methylation profiles of cancer cell lines generated at different study sites (columns) and with different technical platforms (rows). (K) Density plot of Spearman correlation estimates between average gene phosphorylation profiles of cancer cell lines generated at different study sites using RPPA and MS-based technologies. MS/MS-based comparisons could not be performed because MS-based profiling was performed by only one study. (L) Density plot of the Spearman correlation estimates between drug sensitivity profiles of cancer cell lines generated at different study sites using CellTiterGlo (CTG) and Syto60 assays. Syto60/Syto60 comparisons could not be performed because Syto60 based profiling was performed by only one study.

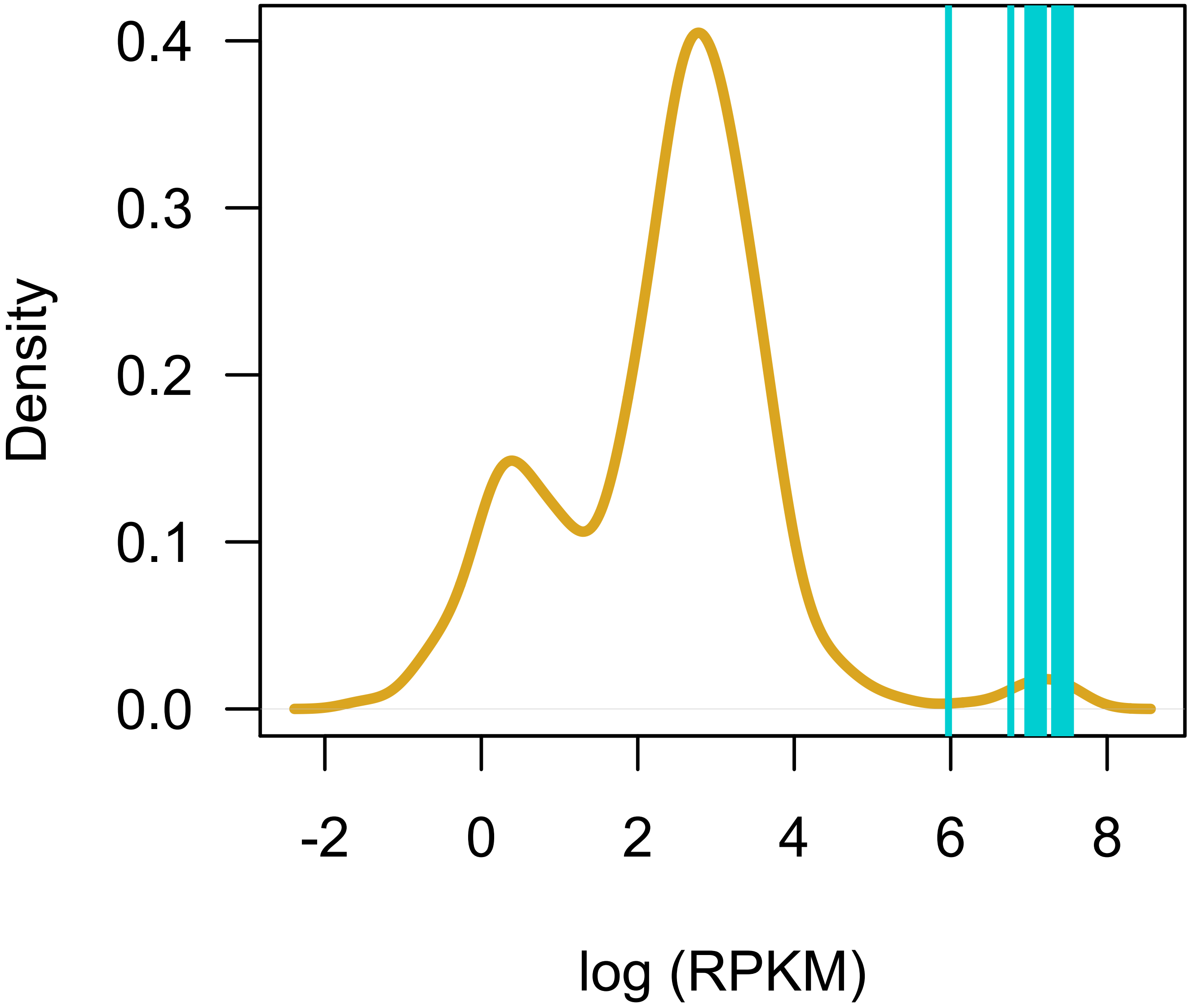

Appendix Figure S10: Distribution of mRNA expression levels of ERBB2 gene in all cancer cell lines profiled in the CCLE dataset. Expression levels of ERBB2 in the breast cancer cell lines that are known to be HER2+ are marked by the turquoise colored vertical lines. As the expression levels of ERBB2 in the HER2+ breast cancer cell lines are towards the right tail, it can be considered that ERBB2 is a CCS gene in these cell lines.

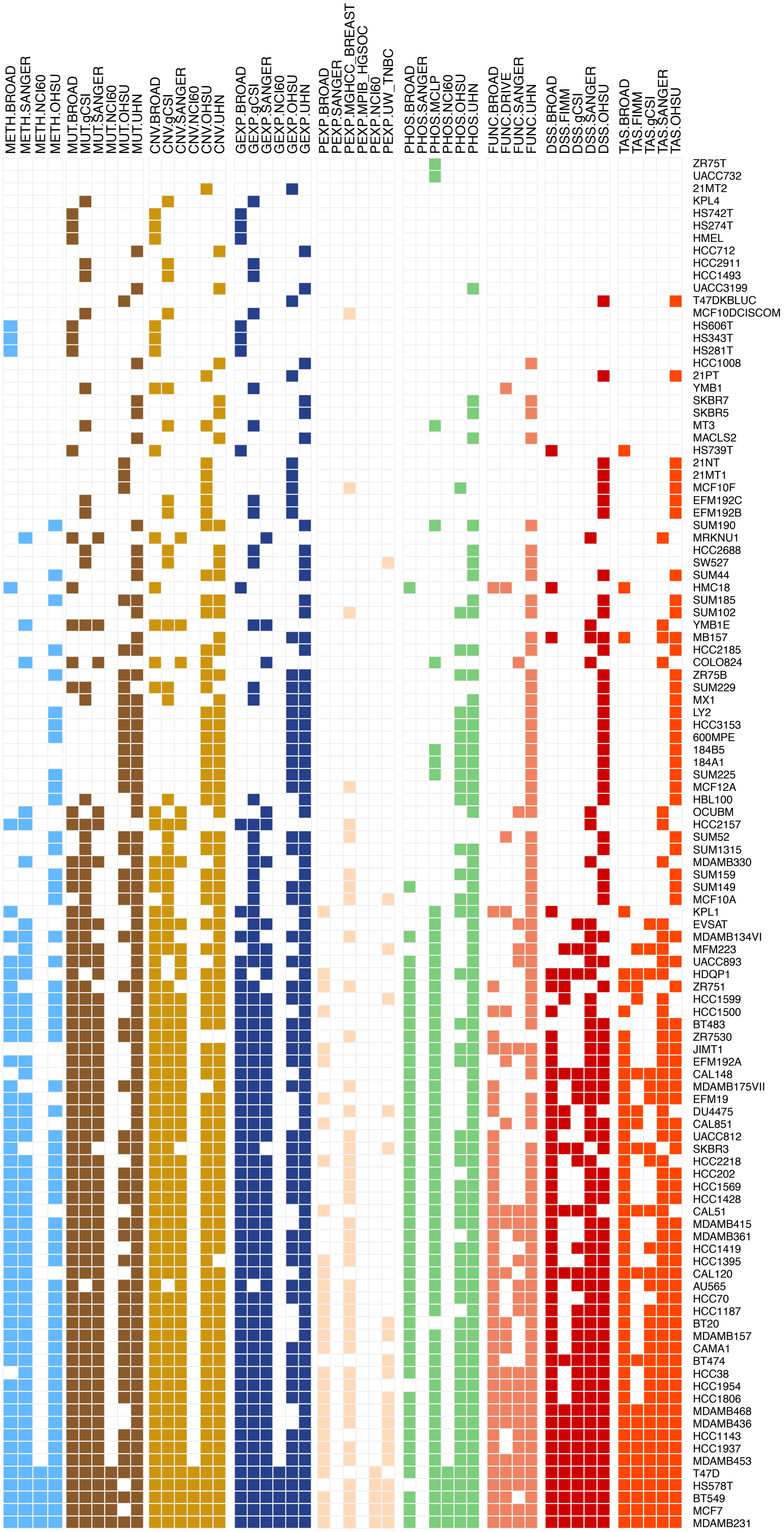

Appendix Figure S11: Data availability of breast cancer cell lines (rows) for each of the modality type from multiple research sites (columns).

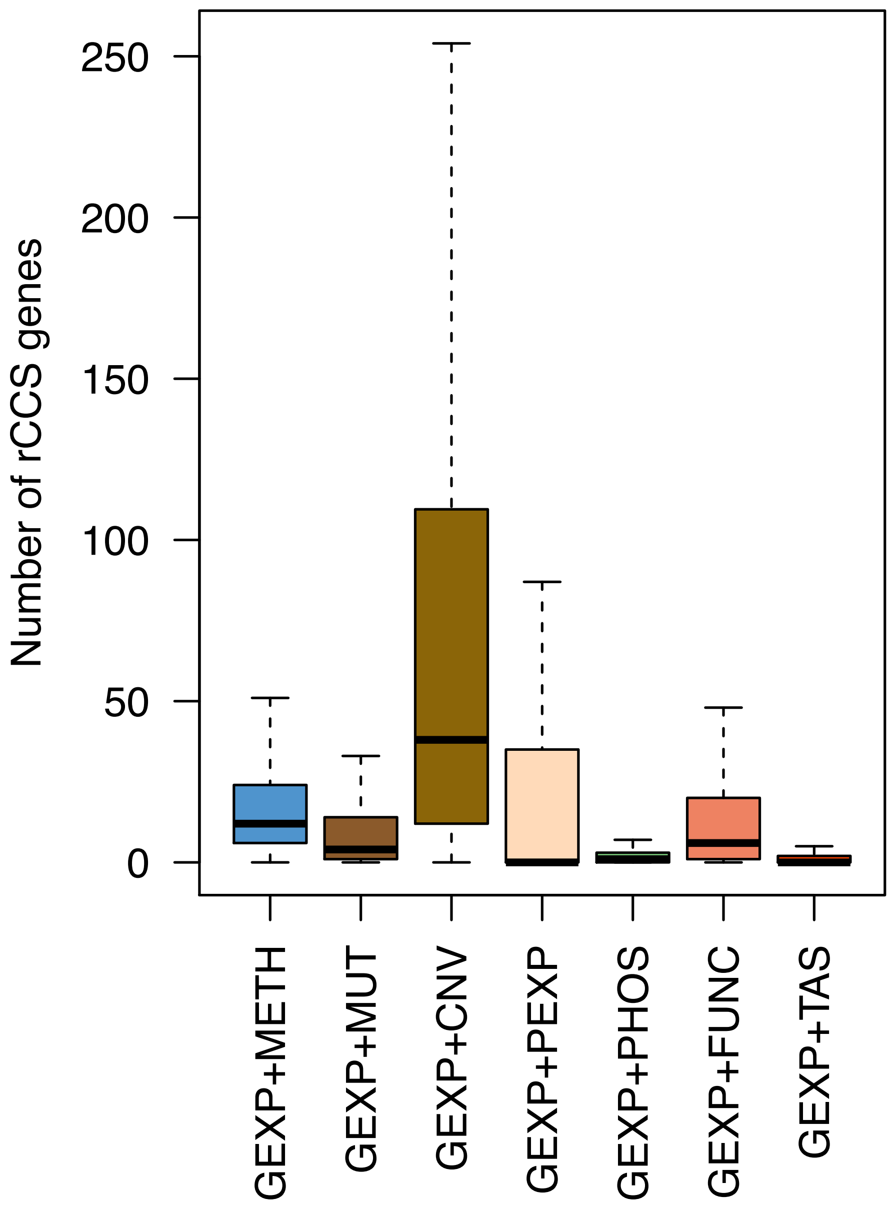

Appendix Figure S12: Number of rCCS genes identified by CLIP for all epithelial cancer cell lines that were supported obligatorily by the GEXP modality in combination with other data modalities. Whiskers represent the range of values, solid line within the box represents the median value.

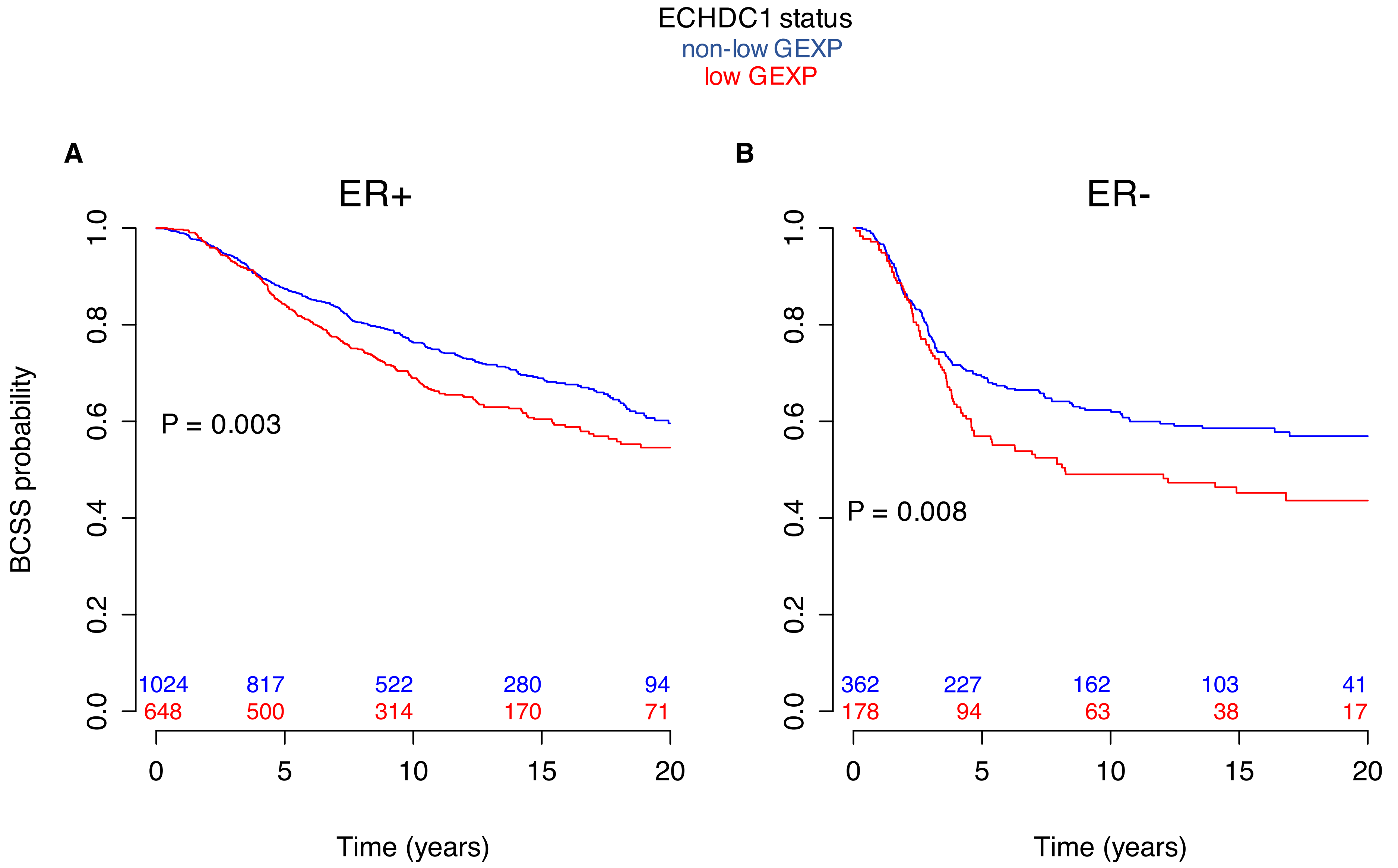

Appendix Figure S13: Breast cancer specific survival (BCSS) analysis based on gene expression and methylation levels status of ECHDC1 in breast cancer patient tumors in the combined Metabric and Oslo cohort datasets. Patients in the low GEXP category class have lower survival probability than those in the non-low GEXP class in the subsets of breast cancer patients defined by the ER status; (A) ER+ patients (n=2873) (B) ER- patients (n=904). Numbers above the x-axis line indicate the number of patients in each group, defined by the color code, at each time point. P-values from age adjusted Cox-proportion hazard model.

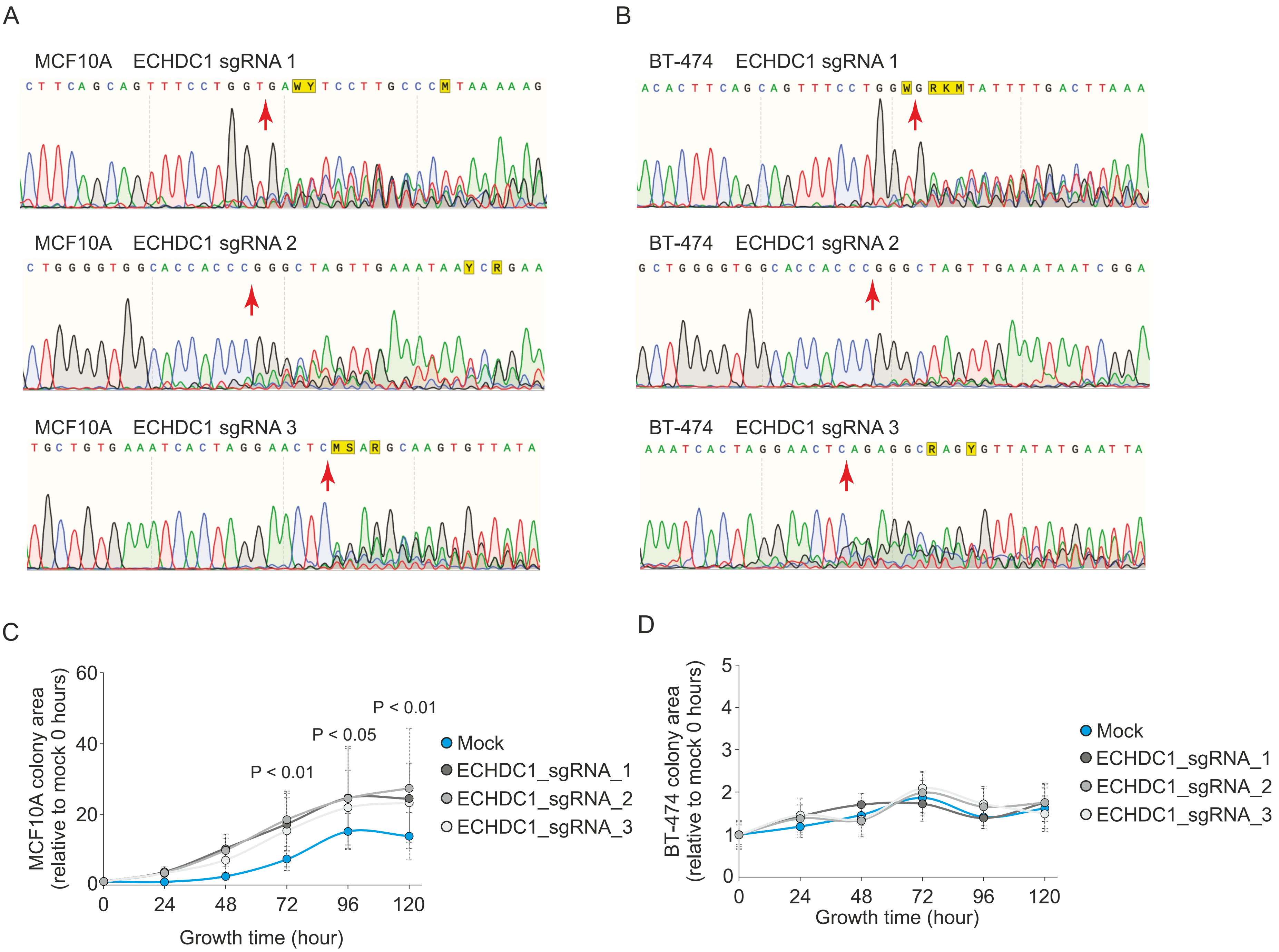

Appendix Figure S14: (A-B) Sanger sequencing results of ECHDC1 sgRNA target regions for MCF10A (A) and BT-474 (B) knock-out cell lines. (C-D) Quantitative assessment of ECHDC1 sgRNA knockout MCF10A and BT-474 colony area in 3D collagen I over 5 days. At 72 hours MCF10A mock versus ECHDC1_sgRNA_2 $P < 0.01$; at 96 hours mock versus ECHDC1_sgRNA_1 and ECHDC1_sgRNA_2 $P < 0.05$; at 120 hours mock (13.8 ± 3.3) versus ECHDC1_sgRNA_1 (24.4 ± 4.9), ECHDC1_sgRNA_2 (27.3 ± 8.5) and ECHDC1_sgRNA_3 (23.3 ± 5.6) $P < 0.01$. Error bars indicate mean ± SEM; n ≥ 10 colonies. Statistical significance was assessed with one-way ANOVA with Tukey's multiple comparison test. See Figure 5C-D for representative light micrographs.

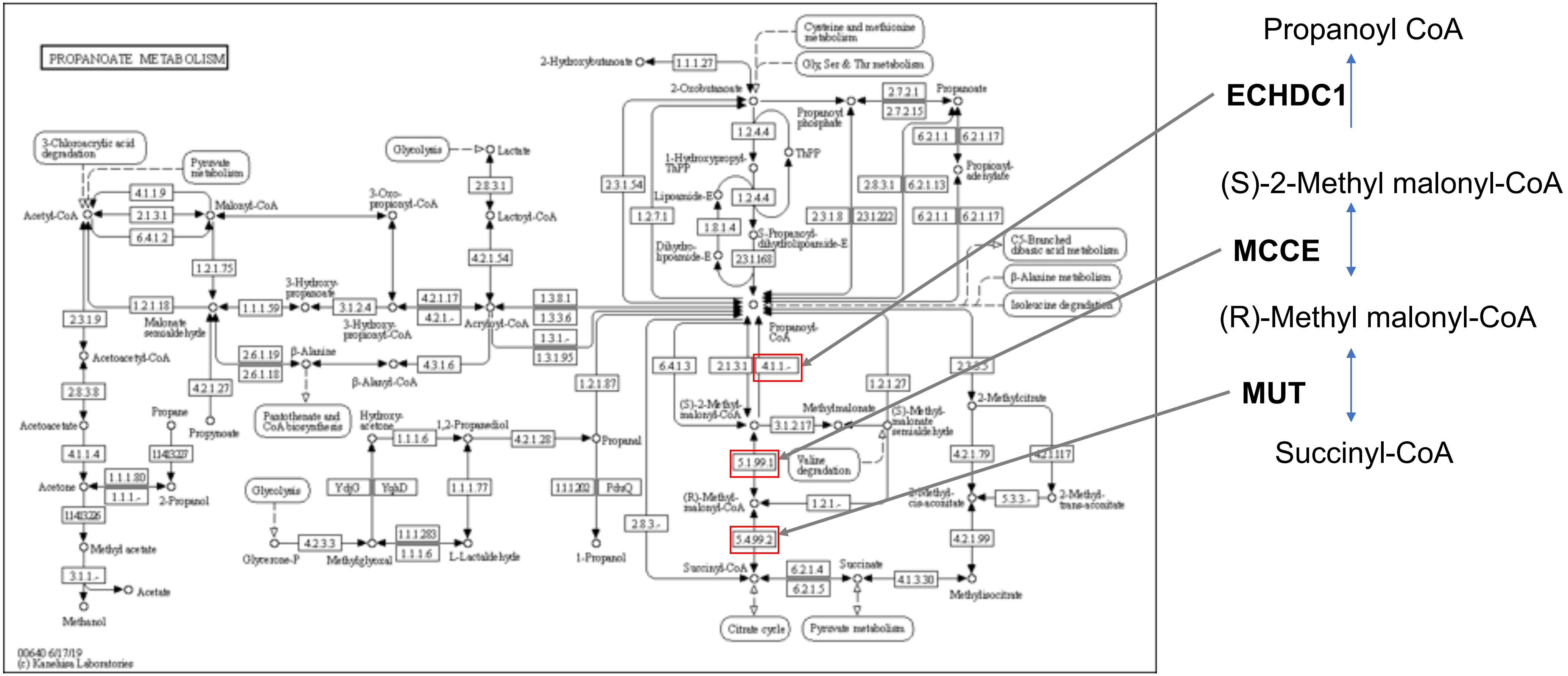

Appendix Figure S15: Propanoate metabolism based on the KEGG database. ECHDC1 and other genes in the pathway are highlighted.

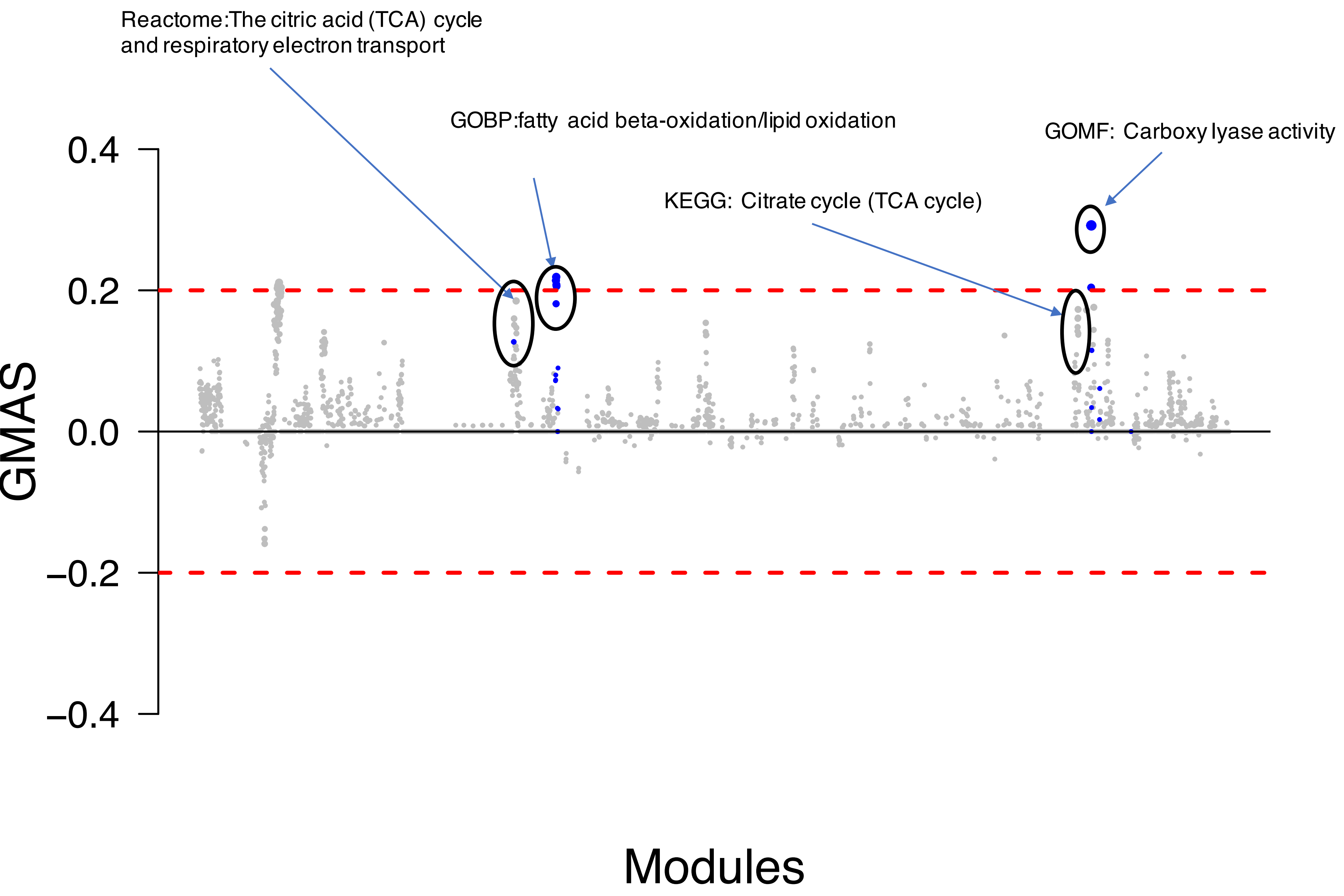

Appendix Figure S16: Gene Module Association Score (GMAS) for modules enriched for co-expression with ECHDC1, calculated based on Gene-Module Association Determination (G-MAD) algorithm, implemented in the GeneBridge toolkit. For details, see Table S3.

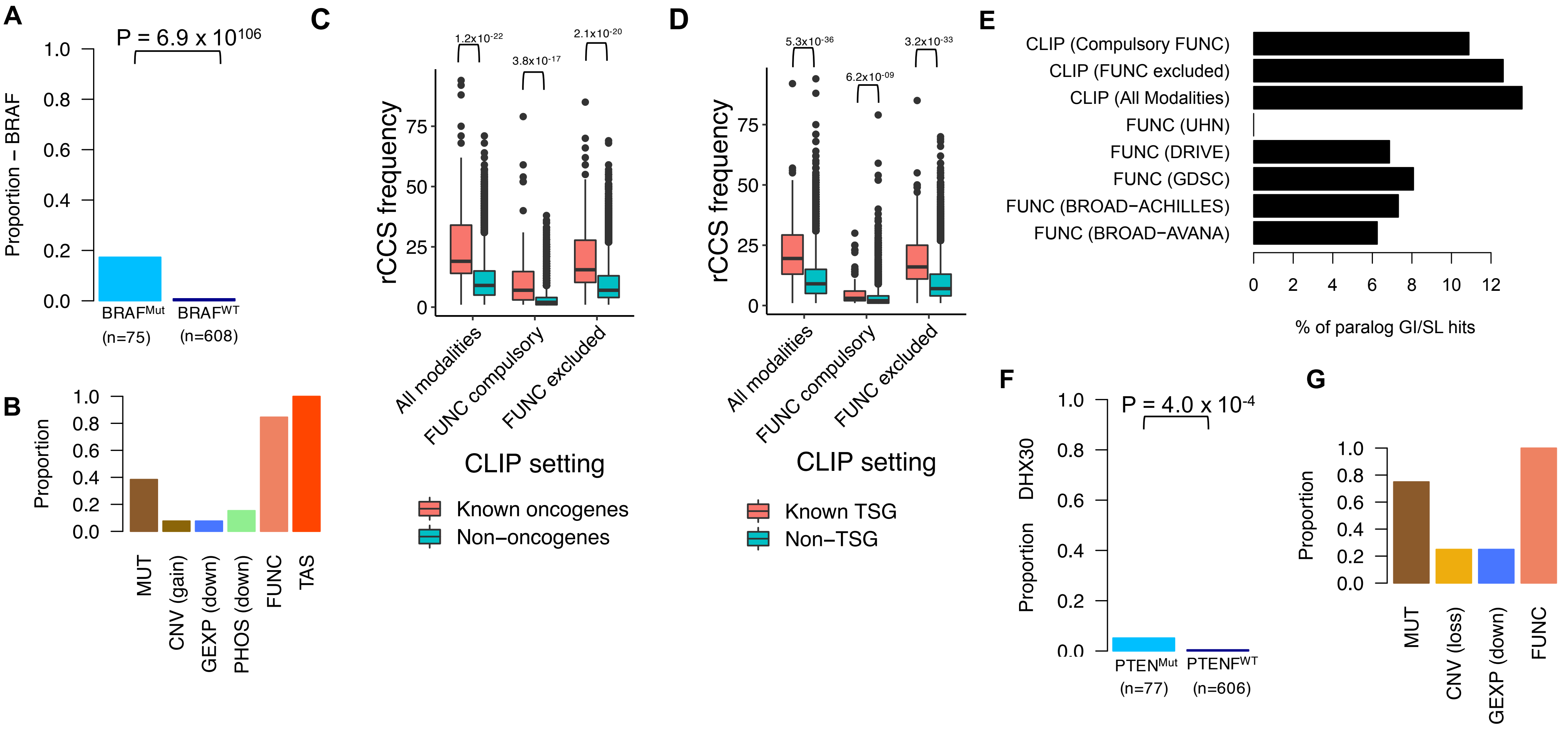

Appendix Figure S17: (A) Proportion of BRAF mutated (Mut) and BRAF wild type (WT) cancer cell lines with BRAF identified as a rCCS gene. P-value was calculated with Fisher's exact test. (B) The modalities that supported the rCCS status of BRAF and the proportion of cell lines having that evidence in the BRAF mutated cell lines. (C-D) Systematic identification of cancer oncogenes (C) and tumor suppressor genes (TSG) (D) specific to epithelial tumors under multiple settings of CLIP run. rCCS genes identified by CLIP are enriched for known cancer drivers compared to non-driver genes, even after excluding the FUNC data modality from the CLIP approach. P-values are calculated with Wilcoxon test. (E) Proportion of SL interactions between paralogous genes identified by CLIP and alternative approaches based on each FUNC dataset (see Materials and Methods for details). (F) Proportion of PTEN mutated (Mut) and PTEN wild type (WT) cancer cell lines in which DHX30 was identified as a rCCS gene. P-value was calculated with Fisher's exact test. (G) The modalities that supported the rCCS status of DHX30 and the proportion of cell lines having that evidence in the PTEN mutated cell lines.

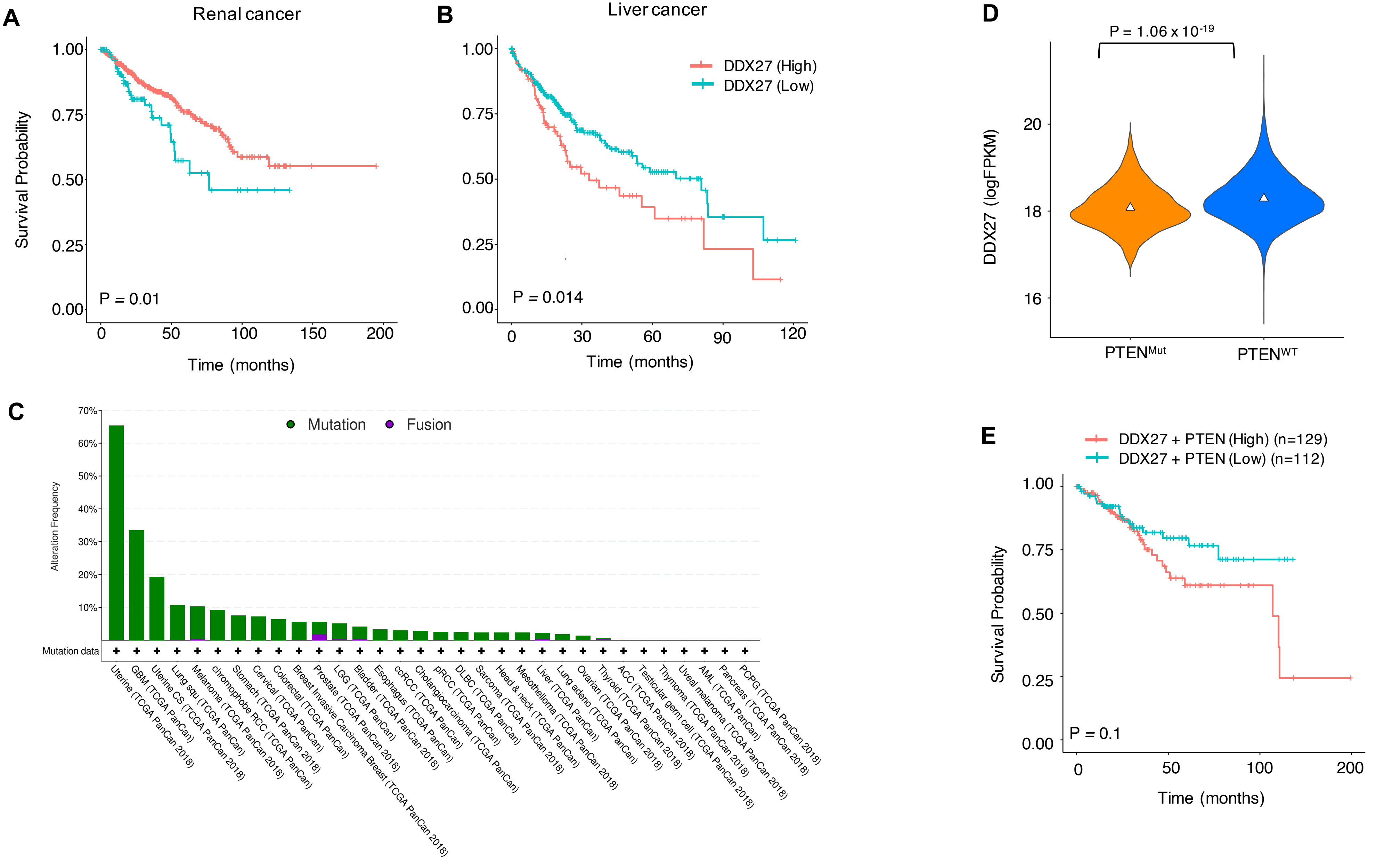

Appendix Figure S18: (A-B) Survival analysis in patients with liver cancer (n=353) and renal cancer (n=275) in the TCGA dataset categorized by mRNA expression levels of DDX27. Expression levels were divided into two classes, high and low, based on mean expression levels of DDX27 (logFPKM=18.27). Patients in the high class showed lower survival probability than those in the low class ($P < 0.05$; log-rank test). (C) Breakdown of mutational frequency of PTEN by cancer tissue type in TCGA Pan-cancer dataset, performed in cBioPortal (D) mRNA expression levels of DDX27 in PTEN mutated (Mut, n=582) and PTEN wild type (WT, n=7012) in TCGA dataset of all epithelial cancer types. P-value was calculated using Wilcoxon test. (E) Survival analysis of in patients with endometrial cancer in TCGA dataset categorized by mRNA expression levels of DDX27 and PTEN. Expression levels were divided into two classes, high and low, based on mean expression levels of DDX27 (logFPKM=18.19) and PTEN (logFPKM=17.11). Patients belonging to high expression category for both genes showed lower survival probability than those in the low expression category. P-values were estimated using the log-rank test.

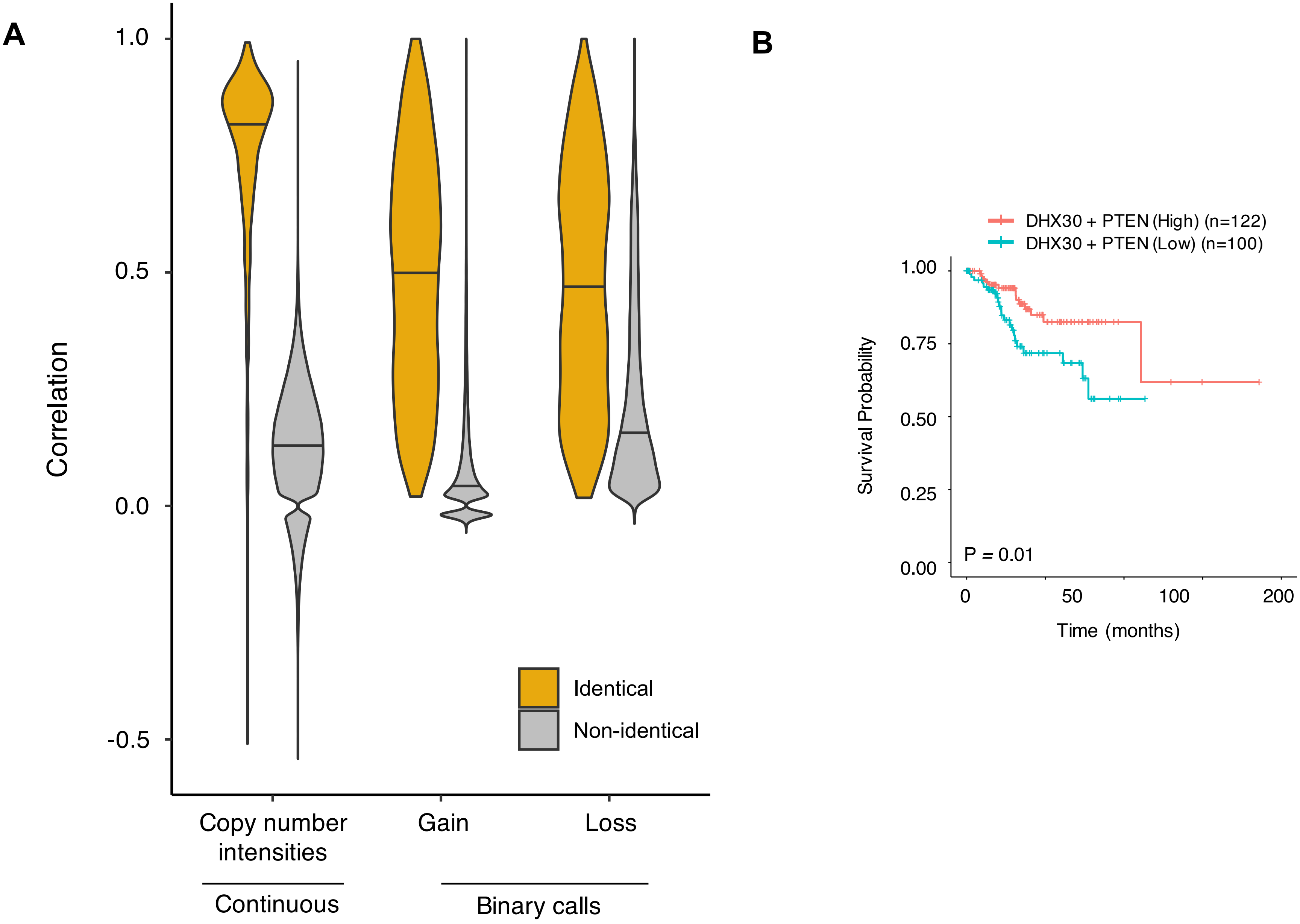

Appendix Figure S19: Correlation of the different types of copy number profiles. Spearman correlation was calculated between identical and non-identical cell lines between datasets from various research sites for continuous copy number calls (reused from Figure 1D). For binarized CNV calls of gains and losses, correlation was estimated by Matthew's correlation coefficient. (B) Survival analysis of in patients with endometrial cancer in TCGA dataset categorized by mRNA expression levels of DHX30 and PTEN. Expression levels were divided into two classes, high and low, based on mean expression levels of DHX30 (logFPKM=18.67) and PTEN (logFPKM=17.11). Patients belonging to high expression category for both genes showed lower survival probability than those in the low expression category. P-values were estimated using the log-rank test.
